# Associations between late pregnancy Dietary Inflammatory Index (DII) and offspring bone mass: a meta-analysis of the Southampton Women’s Survey (SWS) and the Avon Longitudinal Study of Parents and Children (ALSPAC)

**DOI:** 10.1002/jbmr.4623

**Published:** 2022-06-10

**Authors:** Stephen Woolford, Stefania D’Angelo, Giulia Mancano, Elizabeth M Curtis, Shanze Ashai, Nitin Shivappa, James Hébert, Sarah R Crozier, Catherine Phillips, Matthew Suderman, Caroline Relton, Cyrus Cooper, Nicholas C Harvey, the ALPHABET Consortium Investigators, Adrien M Aubert, Jonathan Y Bernard, Ling-Wei Chen, Liesbeth Duijts, Aisling A Geraghty, Wojciech Hanke, Barbara Heude, Cecily C Kelleher, Fionnuala M McAuliffe, John Mehegan, Sara M Mensink-Bout, Kinga Polanska, Elzbieta Trafalska

**Affiliations:** a MRC Lifecourse Epidemiology Centre, University of Southampton, Southampton, UK; b MRC Integrative Epidemiology Unit, Population Health Sciences, Bristol Medical School, University of Bristol, Bristol, UK; c Cancer Prevention and Control Program and Department of Epidemiology and Biostatistics, Arnold School of Public Health, University of South Carolina, Columbia, South Carolina, USA; d Department of Nutrition, Connecting Health Innovations LLC, Columbia, South Carolina, USA; e NIHR Applied Research Collaboration Wessex, Southampton Science Park, Southampton, UK; f School of Public Health, Physiotherapy, and Sports Science, University College Dublin, Belfield, Ireland; g NIHR Southampton Biomedical Research Centre, University of Southampton and University Hospital Southampton NHS Foundation Trust, Southampton, UK; h NIHR Biomedical Research Centre, University of Oxford, Oxford, UK

**Keywords:** osteoporosis, epidemiology, childhood, bone, DXA, E-DII, diet, inflammation, SWS, ALSPAC

## Abstract

Systemic inflammation is associated with reduced bone mineral density and may be influenced by pro-inflammatory diets. We undertook an observational analysis of associations between late pregnancy energy-adjusted Dietary Inflammatory Index (E-DII™) scores and offspring bone outcomes in childhood. E-DII scores (higher scores indicating pro-inflammatory diets) were derived from food frequency questionnaires in late pregnancy in two prospective mother-offspring cohorts: the Southampton Women’s Survey (SWS) and the Avon Longitudinal Study of Parents and Children (ALSPAC). The mean (SD) offspring age at DXA scanning was 9.2 (0.2) years. Linear regression was used to assess associations between E-DII and bone outcomes, adjusting for offspring sex and age at DXA and maternal age at childbirth, educational level, pre-pregnancy BMI, parity, physical activity level and smoking in pregnancy. Associations were synthesised using fixed-effect meta-analysis. Beta coefficients represent the association per unit E-DII increment. In fully adjusted models (total n=5910) late-pregnancy E-DII was negatively associated with offspring whole body minus head bone area (BA: β=-3.68 [95%CI: -6.09, -1.27] cm^2^/unit), bone mineral content (BMC: β=-4.16 [95%CI: -6.70, -1.62] g/unit) and areal bone mineral density (aBMD: β=-0.0012 [95%CI: -0.0020, -0.0004] g.cm^-2^/unit), but there was only a weak association with BMC adjusted for BA (β=-0.48 [95% CI: -1.11, 0.15] cm^2^/unit) at 9 years. Adjustment for child height partly or, for weight, fully attenuated the associations. Higher late pregnancy E-DII scores (representing a more pro-inflammatory diet) are negatively associated with offspring bone measures, supporting the importance of maternal and childhood diet on longitudinal offspring bone health.

## Introduction

Chronic systemic inflammation has been associated with several musculoskeletal outcomes including osteoarthritis, osteoporosis and fragility fractures.^(1–4)^ Dietary components, such as certain carbohydrates and fatty acids, have been found to contribute to such a chronic inflammatory state,^(5)^ which may detrimentally affect long-term health. For example, Western diets rich in red meat, high fat, sugar, dairy products and refined grains have been associated with greater inflammatory load.^(6–8)^ The Dietary Inflammatory Index (DII^®^) was created to quantify the inflammatory potential of diet ^(9)^ and, after the first validation ^(10)^ has now been validated with pro-inflammatory cytokines and other biomarkers in over 40 studies from around the world.^(11)^ Both higher DII^(12)^ and higher energy-adjusted DII (E-DII™) scores (both indicating a more pro-inflammatory diet) have been associated with lower bone mineral density and higher fracture risk in adults.^(13,14)^ Consistent with these musculoskeletal outcomes, higher DII/E-DII score has been associated with a wide range of NCDs, including cardiovascular diseases,^(15,16)^ colorectal cancer^(17)^ and depression.^(18)^

Additionally, higher maternal E-DII during pregnancy has been associated with lower birth weight and shorter birth length,^(19)^ which may have detrimental implications for early-life bone development. These observations, together with well-established links between the early environment and later musculoskeletal development, give rise to the hypothesis that greater dietary inflammatory load in early life, for example *in utero,* may impair bone accrual,^(20)^ which might lead to a reduction in peak bone mass (PBM) achieved in early adulthood,^(21,22)^ with implications for osteoporosis and fracture risk in older age.^(23–25)^

We therefore aimed to investigate the associations between late-pregnancy and early-childhood E-DII and offspring bone outcomes in later childhood in two longitudinal mother-child cohorts within the EU ALPHABET consortium: the Southampton Women’s Survey (SWS) and the Avon Longitudinal Study of Parents and Children (ALSPAC).

## Methods

The ALPHABET consortium is a European Union and national partner organisation-funded collaboration bringing together seven mother-child cohorts across five European countries. Its aim is to investigate the associations between maternal diet quality, dietary inflammatory potential, epigenetic markers, and offspring health, ultimately informing future public health policy. The Southampton Women’s Survey (SWS) and the Avon Longitudinal Study of Parents and Children (ALSPAC) are two UK-based prospective mother-offspring cohort studies contributing data to the ALPHABET consortium, and both investigate the effects of anthropometric, environmental, and genetic characteristics on maternal and offspring health. These two cohorts form the basis of the current analysis because of the availability of childhood DXA data in addition to the E-DII data. The structure and methods of the SWS and ALSPAC cohorts have been described in detail elsewhere.^(26–28)^

In brief, 12,583 non-pregnant women aged 20-34 years were recruited to the SWS between 1998 and 2002 from the general population of Southampton, UK, with no other exclusion criteria. For ALSPAC, pregnant women with an expected delivery date between 1^st^ April 1991 and 31^st^ December 1992 and living in the area of Avon, England, were recruited, with 14,541 women entering the study. For both cohorts, only the first child born during the study time frame was included in further analyses. There was a total of 3158 and 14,062 singleton live births being followed for the SWS and ALSPAC, respectively.

In both cohorts, anthropometry and lifestyle assessments were undertaken at study recruitment, with comparable methodology. Maternal height was measured using a stadiometer and weight with a calibrated digital scale, with BMI being calculated. Research nurses underwent training and regular re-assessment to ensure measurement consistency. Information on parity, educational level, smoking habits and regular physical activity was also gathered via questionnaire at study recruitment. Within the SWS, subsets of mothers from specific GP practices were approached to participate in a bone assessment sub-study and for their child to undergo dual-energy X-ray absorptiometry (DXA) assessment at several timepoints, including 9 years of age. Specific GP practices were chosen to avoid participants being recruited into multiple sub-studies. In ALSPAC, at 9 years all children with known addresses who were still participating in the study were invited to a “Focus @ 9” clinic, with DXA scanning at clinic attendance being offered. In neither study was there a specific inclusion criteria other than the ability to undergo DXA scanning.

The SWS was approved by the Southampton and South West Hampshire Local Research Ethics Committee. Ethics approval for ALSPAC was obtained from the ALSPAC Ethics and Law Committee and the Local Research Ethics Committees. Informed consent for the use of data collected via questionnaires and clinics was obtained from participants following the recommendations of the ALSPAC Ethics and Law Committee at the time. Written consent was obtained from the parent or carer of all participants at each study stage.

### Dietary inflammatory index measurement

The design and development of the original Dietary Inflammatory Index (DII^®^) was published in 2009.^(29)^ Methods were updated about 4 years later, which produced a revised tool addressing shortfalls in the original model, for example standardising units of measurement to global referent values and using a markedly expanded literature.^(30)^ The energy-adjusted dietary inflammatory index (E-DII™) was later created to account for differences in energy intake that could influence inflammatory capacity of the diet and could be related to sex, physical activity, and body size.^(11)^ At this juncture, the E-DII fits the data better (produces greater model explanatory ability and better goodness of fit) than the DII in about 2/3^rd^ instances. The current version of both the DII and E-DII reflect evidence-based scoring systems derived from a comprehensive review yielding around 6500 articles relating dietary parameters to 6 inflammatory markers (IL-1b, IL-4, IL-6, IL-10, TNF-a and C-reactive protein). A total of 1943 studies were found to report on primary associations between these 6 inflammatory markers and 45 food parameters, including 10 whole food items and 35 nutrient measures.^(9)^ Individual food parameters were assigned a positive score (+1) if associated with a pro-inflammatory response, a negative score (-1) if associated with an anti-inflammatory response, or a score of zero if not associated with inflammatory response.^(9)^ In the present study dietary information from participants was converted to an amount consumed per 1,000-kcal and then linked to a regionally representative database with similarly energy-adjusted values. This database provided an overall estimate of mean and standard deviation of energy-standardized intakes for each of the dietary parameters collected for the participant (i.e., nutrients, foods, and other food components) and these data also were adjusted for energy using the density method. By subtracting the mean of the energy-adjusted regionally representative database from the individual’s reported amount and dividing this value by the parameter’s representative standard deviation, z-scores for each dietary parameter were derived. These z-scores were converted to cumulative proportions (i.e., with values ranging from 0 to 1) and then centred by doubling each and subtracting 1. The resulting value was then multiplied by the corresponding nutrient parameter effect score. These food parameter-specific E-DII scores were then summed to yield an overall E-DII score. In this way, an overall E-DII score can be calculated for an individual, with more positive E-DII scores indicating a more pro-inflammatory dietary pattern, and lower E-DII scores indicating a more anti-inflammatory diet. Construct validity has since been assessed in relation to inflammatory biomarkers in over 39 studies.^(10,29,31)^

Full methodology regarding how E-DII scores were calculated for the SWS and ALSPAC has been published previously.^(32)^ Briefly, in both the SWS and ALSPAC maternal E-DII scores were calculated from food frequency questionnaires (FFQ) at late pregnancy follow-up clinics (34 weeks’ gestation in the SWS and 32 weeks’ gestation in ALSPAC). Offspring diet was assessed at 3 years of age, via postal FFQs^(6)^ from which children’s-DII (C-DII) scores were generated^(6)^. A total of 100 food items were measured by FFQ in the SWS (previously validated against 4 day food diary^(33)^) and a total of 43 food items were measured by FFQ in ALSPAC^(34)^, with additional questions about bread, milk, fats and drinks. Of these food items, 24 and 28 dietary parameters were derived to calculate DII from the SWS and ALSPAC, respectively.^(32)^ The specific food/nutrient items considered in each cohort are documented in Supplementary Table 2.^(32)^

### Childhood DXA assessment

In the SWS, the child’s height (without shoes), using a stadiometer (Seca, Birmingham, UK), and weight (without shoes and wearing light clothing), using calibrated digital scales (Seca, Birmingham, UK), were measured. Similar measurements were taken in ALSPAC, using a stadiometer and a Tanita body fat analyser (Tanita Europe BV). The child’s age at the time of DXA assessment was also recorded. A Hologic Discovery DXA scanner was used in the SWS children (Hologic Inc., Bedford, MA, USA), while a Lunar Prodigy DXA scanner was used in the ALSPAC (GE Healthcare, UK). Whole-body scans were obtained, generating data on bone indices. Coefficients of variation for whole body BMD were 0.75% and 0.84% in the SWS and ALSPAC, respectively. DXA scans were reviewed and those with excessive movement or clothing artefacts were omitted from the analysis. In the SWS, 1024 children underwent DXA assessment at 9 years, with 990 having useable images for analysis. In ALSPAC, 7722 children underwent 9-year DXA assessment, with 7333 having useable images for analysis. Bone outcomes of interest obtained directly from the DXA assessment were whole-body bone area (BA), whole-body bone mineral content (BMC) and whole-body areal bone mineral density (aBMD).

### Statistical analysis

All baseline characteristics were checked for normality of distribution and described using mean (SD), median (IQR) or number (%), as appropriate. Whole body minus head DXA data were analysed.^(35)^ As a further measure to correct for body size, BMC adjusted for BA was used. Outcomes of interest were initially related to late pregnancy E-DII using univariable linear regression analyses. Based on prior literature, biological plausibility and availability across both cohorts, the following covariates were considered in fully adjusted models: offspring sex and age at DXA scanning and maternal age at child’s birth, educational level (A level-equivalent qualification or above in both cohorts), pre-pregnancy BMI, parity, physical activity level (hours of strenuous activity per week in the SWS and regular physical activity at least once a week in ALSPAC) and smoking status in pregnancy. In further exploratory analyses the potential mediating effect of offspring height or weight was investigated. Additional exploratory analyses used the childhood 3-year C-DII measure as the exposure. Finally, because obesity has relevance for both systemic inflammation and bone health, and is clearly linked to diet, we examined whether there were any interactions between maternal E-DII and BMI on offspring bone outcomes. Beta coefficients generated represent increase in the bone outcome of interest per unit greater E-DII score.

Analyses were carried out in each study separately and a meta-analysis of 8–9-year SWS and ALSPAC results was then performed. Heterogeneity of effect estimates from the two studies was assessed by the Cochran’s Q-statistic and quantified by the *I*^2^ statistic.^(36)^ As there was no statistical evidence of heterogeneity (p>0.1), the effects were combined in a fixed-effects meta-analysis model^(36)^ to estimate the pooled effect of late pregnancy and 3-year offspring DII on bone outcomes at 9 years. Forest plots were used as a graphical display of the results of the meta-analysis. Stata V15.1 (StataCorp LP, College Station, TX, USA) was used for all analyses.

## Results

### Characteristics of mothers and offspring

In the SWS, 931 mothers had E-DII calculated at 34 weeks’ gestation and offspring DXA data at 9 years. Their baseline characteristics are summarised in Table 1. The mean (SD) age at delivery was 30.7 (3.8) years, with 48.6% of mothers being multiparous; 61.5% had a senior school high school (A level-equivalent) qualification or above and 15.1% smoked during pregnancy; 13.7% were obese pre-pregnancy; and 65.4% reported any strenuous physical activity each week. The mean (SD) age of offspring at DXA assessment was 9.2 (0.2) years. Approximately half of all offspring were male. Mean (SD) E-DII score was 0.60 (1.46) for mothers and C-DII -0.04 (1.06) for offspring at age 3 years. Compared with individuals who did not take part in the DXA follow-up, those who did were more likely to have a higher level of education and less likely to smoke during pregnancy. All other characteristics were similar across the groups (Supplementary Table 3a).

In ALSPAC, 6334 mothers had E-DII calculated at 32 weeks’ gestation and offspring who underwent DXA scanning at 9 years. Their baseline characteristics are summarised in Table 2. The mean (SD) age at delivery was 29.2 (4.5) years. Most (53.8%) mothers were multiparous, 33.1% had at least an A level-equivalent qualification, and 14.4% smoked during pregnancy. Mean (SD) pre-pregnancy BMI was 22.9 (3.7) kg/m^2^ and 68.0% of participants reported regular physical activity at least once a week. 48.9% of children were male and the mean (SD) age at DXA scanning was 9.9 years (0.3). Mean (SD) E-DII was 0.37 (1.80) for mothers and C-DII 0.53 (1.29) for offspring. Compared with mothers whose child did not take part in the DXA follow-up, the mothers included in this study had lower E-DII score, were more likely to have at least an A level and to be slightly taller (Supplementary Table 3b).

The participant flow through the two cohorts is summarised in Supplementary Figure 1.

### Maternal late pregnancy E-DII, offspring 3-year C-DII and offspring bone outcomes in SWS and ALSPAC

Associations between either maternal late pregnancy E-DII and offspring bone outcomes are presented for both the SWS and ALSPAC in Table 3, in unadjusted and fully adjusted models. The general pattern is one of greater E-DII (indicating a more pro-inflammatory dietary pattern) associated with poorer bone outcomes (BA, BMC and aBMD), supported by greater statistical evidence in the ALSPAC cohort, consistent with the much larger sample size. In general, associations were robust to adjustment for confounding factors, and in exploratory analyses associations with bone outcomes appeared similar for offspring 3-year C-DII (Supplementary Table 3). There was no evidence of an interaction between maternal E-DII and BMI on offspring bone outcomes. Further adjustment for either childhood height (Supplementary Table 4) or weight (Supplementary Table 5) led to partial or full attenuation of the associations respectively. There was no evidence of associations between either maternal pregnancy E-DII or childhood C-DII and childhood BMC adjusted for BA. As an illustration of the effect size, comparing offspring of mothers in the top compared with the bottom quarter of E-DII distribution in ALSPAC, BMC was on average 15 g greater, representing a mean difference of 0.08 SD.

### Meta-analysis of 9-year bone outcomes

**Figure 1:**
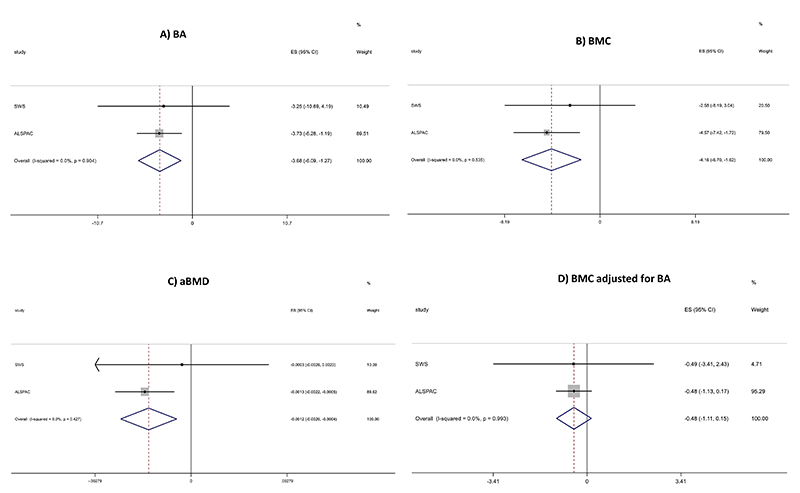
Meta-analysis of associations between late pregnancy E-DII and 9-year offspring bone outcomes in the SWS and ALSPAC cohorts.

Associations between maternal late pregnancy E-DII and 9-year offspring bone outcomes in the SWS and ALSPAC cohorts are presented using forest plots in Figure 1. In fully adjusted models late pregnancy E-DII was negatively associated with offspring BA, BMC and aBMD at 8-9 years, with weaker negative associations apparent for BMC adjusted for BA. In further exploratory analyses, associations between childhood 3-year C-DII score and 9-year bone outcomes in the SWS and ALSPAC cohorts are presented using forest plots in Supplementary Figure 2. In fully adjusted models, there was evidence of marginally greater associations with bone outcomes compared with maternal late pregnancy E-DII score as the exposure for BA, BMC, and aBMD. Again, associations with BMC adjusted for BA were less robust than with the other three bone measures.

## Discussion

To our knowledge, this is the first study to demonstrate associations between consumption of a pro-inflammatory diet during pregnancy or early childhood and offspring bone measures. Higher late pregnancy levels of pro-inflammatory dietary components were associated with lower offspring bone measures at 9 years. The associations were much weaker for BMC adjusted for BA than for BA, BMC or aBMD, with associations at least partly explained by child height and weight. Additionally, a more pro-inflammatory diet at 3 years of age was negatively associated with offspring BA and BMC at 9 years. Thus, overall, our findings support the notion that greater exposure to a more pro-inflammatory diet in early life is associated with poorer bone health in childhood.

We have not been able to identify any prior evidence linking childhood bone accrual with either maternal diet-associated inflammation during pregnancy or via the child’s own diet earlier in postnatal life. However, other work has previously associated DII score in pregnancy and early childhood with a range of other detrimental health outcomes, including reduced offspring birthweight and greater childhood adiposity^(37)(38–40)^, including within the larger ALPHABET consortium.^(19)(32)^ Our findings augment the existing evidence base by demonstrating persistent negative associations between pro-inflammatory diet in pregnancy and childhood with offspring bone mass. Furthermore, although data regarding the musculoskeletal sequelae of elevated DII in pregnancy and early life are somewhat limited, our findings are consistent with those observed at older ages, when DII or other markers of systemic inflammation are associated negatively with measures of bone health.^(1–4,12–14)^ However, several of the studies mentioned above have examined DII, as opposed to E-DII, during pregnancy and early childhood; although DII and E-DII are scored similarly and scaled identically,^(11)^ direct comparison to our results should last be undertaken with this caveat in mind.

Whilst causality cannot be inferred from our results due to the observational nature of this study, several mechanisms have been proposed by which systemic inflammation during pregnancy may detrimentally affect fetal growth and, by extension, offspring bone health. Raised levels of inflammatory cytokines^(41)^ may directly transfer across the placenta to the fetus and subsequently impair fetal development.^(42)^ Maternal inflammation may also lead to local inflammation of the placenta, impairing its ability to facilitate maternal-fetal nutrient transfer.^(43,44)(45)^ Although the mother-offspring associations were partly attenuated through adjustment for offspring height, consistent with the much-attenuated associations with BMC for BA, it was apparent that the relationship was not entirely explained through linear growth. Adjustment for child’s bodyweight largely removed the associations, and given that both lean and fat mass tend to be positively related to bone measures, this raises the possibility that the bone relationships might be secondary to differences in lean or fat mass. A further possibility which arises from the inverse association between pro-inflammatory diets and other measures of a healthy diet (discussed in more detail below), relates to the greater calcium intake associated with a healthy diet.^(46)^ Greater calcium intake did not appear to be pro-inflammatory in the studies underpinning the genesis of the E-DII,^(30)^ but a less pro-inflammatory diet might be associated with better bone health via greater calcium intake through the inverse association between E-DII and healthy dietary patterns.^(46)^ However, the evidence linking maternal calcium intake during pregnancy and offspring bone development is limited, and indeed even calcium intake during childhood is inconsistently associated with bone mineral density.^(47,48)^ Given that vitamin D intake is part of the E-DII, it is difficult to disaggregate its effect as part of the score from any other potential contribution to the associations.

In this collaborative work, cognisant of the complexity and limitations of nutrition research, as documented in a recent position paper,^(49)^ we studied two large and well-characterised longitudinal cohorts, with “gold-standard” measurements of offspring bone indices. However, despite these strengths, there are several limitations that should be considered in the interpretation of results from this study. Firstly, the data produced by the DXA assessment of children can be affected by their lower BMD when compared with adults, and the greater likelihood of children moving during scanning. To allow for this, specific paediatric software was used in both cohorts at all time points, which minimises the loss of edge detection, and images with excessive movement artefacts were excluded from the analysis.^(50)^ Secondly, whilst the SWS and ALSPAC are largely comparable cohorts in terms of methodology and setting, the characteristics of their participants differed in several ways. Specifically, mothers in ALSPAC were less likely to have an A level equivalent qualification and displayed lower mean DII, and offspring in ALSPAC had greater mean E-DII, when compared to the SWS. Undertaking analysis within each cohort and synthesising by meta-analysis should mitigate any potential impact of these differences in the associations observed. Thirdly, whilst E-DII has been extensively validated against a wide range of inflammatory biomarkers in a variety of settings,^(10,29,31,51)^ no internal validation against biomarkers taken from participants within the SWS or ALSPAC was able to be performed. Fourthly, as in virtually all observational studies, diet was self-assessed, which introduces the potential for information bias. Fifthly, there is limited validation of the C-DII measure in younger children, with the previous validation study using C-DII in rather older children aged 6-14 years. The tracking of DII over age is not well characterised and so the relevance of the 3-year measure for bone health at 9 years is uncertain. Hence, we view the associations between the childhood measures as intriguing, but clearly requiring further replication. Sixthly, we were not able to broaden our study to the remaining ALPHABET cohorts as DXA measures at a comparable age were not available for those children. Seventhly, it was not appropriate in this setting to discern an E-DII threshold above which a diet might be regarded as “unhealthy” for bone outcomes. Whilst defining a threshold might be a helpful concept for clinical and public health practice, it would clearly require replication in other cohorts across diverse populations. Finally, we did not have longitudinal repeated measures of diet in mothers or children, and common to observational designs, the potential for residual confounding will always remain. Thus, future studies in independent cohorts including consistent longitudinal assessments of diet are warranted to confirm these findings.

If our observed associations persist into the later lifecourse, there are implications for musculoskeletal health in adulthood. Early bone accrual during childhood is an important contributor to peak bone mass, achieved by approximately the third decade of age.^(21,52)^ Therefore, it is conceivable that E-DII in early life might impact the magnitude of an individual’s peak bone mass with implications for the onset of osteoporosis^(23)^ and increased hip fracture risk^(24)^ in later life. The effect size observed in our analysis is relatively modest, with a mean difference of 0.08 SD in childhood BMC between the bottom and top quarters of the distribution of maternal E-DII. However, the very strong relationship between bone mass and fracture risk suggests that, if maintained, it is still likely to be relevant at the population level.^(53,54)^ For example, a recent modelling analysis has demonstrated that even a modest 0.1 SD increase in the BMD distribution across a population may reduce the overall burden of hip fracture by around 7%.^(55)^ Given that there are over 320,000 hip fractures annually in the USA,^(56)^ although such extrapolation is speculative, it demonstrates that a relatively small difference has the potential for substantial change to the burden of disease in a large population. Thus, although direct clinical implementation of these findings would be currently inappropriate based on this initial analysis, the results in a general sense point towards potential benefits for foods associated with low dietary inflammatory potential. This notion is consistent with the wider observation of inverse relationships between dietary inflammatory load and markers of a healthy diet with correlations between scores around -0.5 to -0.7.^(57–64)^ Indeed, Western diets rich in red meat, high fat, sugar, dairy products and refined grains have been associated with greater inflammatory load.^(6–8)^ Such findings support the importance findings using other approaches to dietary evaluation, such as the “prudent” dietary pattern derived in the SWS mothers, characterising a healthy diet as one based on high consumption of fruit and vegetables, high-fibre and low intake of saturated fats.^(46,65) (30,46–48)^

In summary, we have shown that diets with a greater inflammatory component, measured in both late pregnancy and in offspring childhood, are associated with lower bone mineral density in the offspring at nine years old, with associations at least partly mediated through height and/or bodyweight. These observations further support the importance of maternal and early childhood diet on the longitudinal bone health of offspring, which may contribute to later musculoskeletal disease and fracture risk in older age.

## Supplementary Material

Suppl

## Figures and Tables

**Table 1:** SWS maternal and offspring characteristics

A) Dietary Inflammatory Index		
	n	
Maternal, 34 weeks’ gestation (E-DII)	931	Mean 0.48 (1.47); median 0.54 (-0.52,1.53)
Offspring, 3 years (C-DII)	800	Mean 0.23 (1.47); median -0.03 (-0.76,0.67)

B) Maternal characteristics		
	n^1^	
Age at delivery (years)	931	30.8 (3.7)
Parity (≥ primiparous)	931	442 (47.5)
Educational level (≥ A level)	929	587 (63.2)
Smoked during pregnancy	930	115 (12.4)
Height (cm)	927	163.6 (6.4)
Pre-pregnancy weight (kg)	924	67.3 (13.2)
Pre-pregnancy BMI	923	
< 18.5 (Underweight)		12 (1.3)
18.5 - 25 (Normal)		525 (56.9)
25 - 30 (Overweight)		264 (28.6)
> 30 (Obese)		122 (13.2)
Hours/week of strenuous physical activity (>0)	928	608 (65.5)

C) 9-year offspring characteristics		
	n^2^	
Sex (male)	969	487 (50.3)
Age at scan (years)	969	9.2 (0.2)
Height (cm)	969	135.6 (6.0)
Weight (kg)	966	31.2 (6.4)
BA (cm^2^)	969	1124.3 (157.8)
BMC (g)	969	724.8 (120.4)
aBMD (g/cm^2^)	969	0.64 (0.06)
BMC adjusted for BA (g)	969	725.2 (65.3)

Data are mean (SD), median (IQR) or number (%).

1 Numbers are mothers with 34 weeks’ gestation DII calculated and offspring who underwent DXA assessment at 8 years

2 Numbers are offspring with 3-year DII calculated and BMC available at 8-9 years.

Note that numbers of individuals missing values for descriptive variables are summarised in Supplementary Table 1.

**Table 2:** ALSPAC maternal and offspring characteristics

A) Dietary Inflammatory Index		
	n	
Maternal, 32 weeks’ gestation (E-DII)	6334	0.37 (1.80)
Offspring, 3 years (C-DII)	5710	0.53 (1.29)
B) Maternal characteristics		
	n^1^	
Age at delivery (years)	6103	29.2 (4.5)
Parity (≥ primiparous)	6194	3331 (53.8)
Educational level (≥ A level)	6331	2096 (33.1)
Smoked during pregnancy	5924	852 (14.4)
Height	5768	164.2 (6.6)
Pre-pregnancy weight	5768	61.8 (10.7)
Pre-pregnancy BMI	5826	22.9 (3.7)
Regular physical activity at least once a week	6010	4090 (68.0)
C) 9-year offspring characteristics		
	n^2^	
Sex (male)	5710	2808 (49.2)
Age at scan (years)	5710	9.9 (0.3)
Height	5311	139.5 (6.2)
Weight	5311	34.6 (7.2)
BA (cm^2^)	5710	1136.5 (162.7)
BMC (g)	5710	890.6 (182.5)
aBMD (g/cm^2^)	5710	0.78 (0.05)
BMC adjusted for BA (g)	5710	894.4 (39.7)

Data are mean (SD), median (IQR) or number (%).

1 Numbers are mothers with 34 weeks’ gestation E-DII calculated and offspring who underwent DXA assessment at 9 years of age.

2 Numbers are offspring with 3-year C-DII calculated and BMC available at that time point.

**Table 3:** Associations between maternal late pregnancy (34 weeks) E-DII and offspring bone outcomes at 8-9 years in the SWS or ALSPAC

Late pregnancy E-DII (units)								
	Unadjusted				Adjusted ^1^			
**SWS**	n	β	SE	p	n	β	SE	p
BA (cm^2^)	931	-2.49	3.53	0.48	917	-3.25	3.80	0.39
BMC (g)	931	-1.45	2.69	0.59	917	-2.58	2.86	0.37
aBMD (g/cm^2^)	931	0.0003	0.0012	0.80	917	-0.0003	0.0013	0.84
BMC for BA (g)	931	0.14	1.44	0.92	917	-0.49	1.49	0.74

	Unadjusted				Adjusted ^1^			
**ALSPAC**	n	β	SE	p	n	β	SE	p
BA (cm^2^)	**6334**	**-2.22**	**1.14**	**0.05**	**4993**	**-3.73**	**1.29**	**<0.01**
BMC (g)	**6334**	**-2.95**	**1.28**	**0.02**	**4993**	**-4.57**	**1.45**	**<0.01**
aBMD (g/cm^2^)	**6334**	**-0.0011**	**0.0004**	**<0.01**	**4993**	**-0.0013**	**0.0004**	**<0.01**
BMC for BA (g)	6334	-0.52	0.28	0.06	4993	-0.478	0.33	0.15

Table shows regression coefficient and standard error from univariable and multivariable linear regression analyses. Outcomes are whole body measurements, without heads. Results with p≤0.05 shown in bold.

1 Adjusted for offspring sex and age at DXA and maternal age at childbirth, educational level, pre-pregnancy BMI, parity, physical activity level and smoking in pregnancy status.
