# Supplementary material for: Associations Between Late Pregnancy Dietary Inflammatory Index (DII) and Offspring Bone Mass: A Meta‐Analysis of the Southampton Women's Survey (SWS) and the Avon Longitudinal Study of Parents and Children (ALSPAC)
Source: J Bone Miner Res. 2022 Jul 4;37(8):1511–9. doi: 10.1002/jbmr.4623 (PMC9542867; doi:10.1002/jbmr.4623)
Supplement: Supplementary file 1 [file JBMR-37-1511-s001.docx]

**Associations between late pregnancy and early childhood Dietary Inflammatory Index (DII) and offspring bone mass: a meta-analysis of the Southampton Women’s Survey (SWS) and the Avon Longitudinal Study of Parents and Children (ALSPAC)**

S J Woolford ^a^, S D’Angelo ^a^, G Mancano ^b^, E M Curtis ^a^, S Ashai ^a^, N Shivappa ^c^, J R Hébert ^c, d^, S R Crozier ^a,e^, C M Phillips ^f^, M Suderman ^b^, C L Relton ^b^, C Cooper^ a, g, h^, N C Harvey^ a, g ^and the ALPHABET Consortium Investigators

**Online Supplementary material**

**ALSPAC cohort description**

Pregnant women resident in Avon, UK with expected dates of delivery 1st April 1991 to 31st December 1992 were invited to take part in the study. The initial number of pregnancies enrolled is 14,541 (for these at least one questionnaire has been returned or a “Children in Focus” clinic had been attended by 19/07/99). Of these initial pregnancies, there was a total of 14,676 foetuses, resulting in 14,062 live births and 13,988 children who were alive at 1 year of age. 

When the oldest children were approximately 7 years of age, an attempt was made to bolster the initial sample with eligible cases who had failed to join the study originally. As a result, when considering variables collected from the age of seven onwards (and potentially abstracted from obstetric notes) there are data available for more than the 14,541 pregnancies mentioned above. The number of new pregnancies not in the initial sample (known as Phase I enrolment) that are currently represented on the built files and reflecting enrolment status at the age of 24 is 913 (456, 262 and 195 recruited during Phases II, III and IV respectively), resulting in an additional 913 children being enrolled. The phases of enrolment are described in more detail in the cohort profile paper and its update (see footnote 4 below). The total sample size for analyses using any data collected after the age of seven is therefore 15,454 pregnancies, resulting in 15,589 foetuses. Of these 14,901 were alive at 1 year of age.  A 10% sample of the ALSPAC cohort, known as the Children in Focus (CiF) group, attended clinics at the University of Bristol at various time intervals between 4 to 61 months of age. The CiF group were chosen at random from the last 6 months of ALSPAC births (1432 families attended at least one clinic). Excluded were those mothers who had moved out of the area or were lost to follow-up, and those partaking in another study of infant development in Avon.

**Supplementary figure 1:** Flowchart of study participation in a) SWS and B) ALSPAC 

a) **SWS**

Non-pregnant women recruited to the SWS

**N=12,583**

Children with whole body BMC at 9 years and 3-year E-DII

**N=969**

Children with whole body BMC at 9 years and LP E-DII

**N=931**

Children who undertook DXA assessment at 9 years and have BMC measurement

**N=990**

Singleton pregnancies

**N=3,156**

b) **ALSPAC**

Pregnant women recruited for ALSPAC

**N=15,656**

Singleton pregnancies

**N=15,104**

Mothers whose child undertook DXA assessment at 9  

**N=7136**

Mothers with prenatal DII 

**N=6334**

Children with 3-year E-DII

**N=5710**

**Supplementary Table 1:** Numbers of missing items for covariates

| **Covariate** | **SWS (total n=990)** | **ALSPAC (total n=6334)** |
| --- | --- | --- |
| Offspring sex | 0 | 0 |
| Offspring age at DXA | 0 | 0 |
| Maternal age at childbirth | 0 | 231 |
| Maternal educational level | 2 | 3 |
| Maternal pre-pregnancy BMI | 9 | 508 |
| Maternal parity | 0 | 140 |
| Maternal physical activity | 3 | 324 |
| Maternal smoking in pregnancy | 9 | 410 |

**Supplementary Table 2:** Food/nutrient items included in derivation of SWS and/or ALSPAC E-DII scores

| **Food parameters for E-DII generation** | **ALSPAC** | **SWS** |
| --- | --- | --- |
| Beta Carotene | ✓ | ✓ |
| Folic Acid | ✓ | ✓ |
| Vitamin A | ✓ | ✓ |
| Alcohol | ✓ | ✓ |
| Carbohydrate | ✓ | ✓ |
| Cholesterol | ✓ | ✓ |
| Fat | ✓ | ✓ |
| Fiber | ✓ | ✓ |
| Iron | ✓ | ✓ |
| Magnesium | ✓ | ✓ |
| MUFA | ✓ | ✓ |
| Niacin | ✓ | ✓ |
| Protein | ✓ | ✓ |
| PUFA | ✓ | ✓ |
| Riboflavin | ✓ | ✓ |
| Saturated fat | ✓ | ✓ |
| Selenium | ✓ | - |
| Thiamin | ✓ | - |
| Vitamin B 12 | ✓ | ✓ |
| Vitamin B 6 | ✓ | ✓ |
| Vitamin C | ✓ | ✓ |
| Vitamin D | ✓ | ✓ |
| Vitamin E | ✓ | ✓ |
| Zinc | ✓ | ✓ |
| Garlic | - | - |
| Onion (in grams) | - | ✓ |
| Tea (in grams) | ✓ | ✓ |
| Caffeine | ✓ | - |
| Omega 3 | ✓ | - |
| Omega 6 | - | - |
| Trans Fat | ✓ | - |

The table shows included foods/nutrients (✓) from the complete set considered across the ALPHABET cohorts.

PUFA, polyunsaturated fatty acids; MUFA, monounsaturated fatty acid

**Supplementary Table 3a:** Baseline characteristics of mothers and children for those not undergoing offspring DXA at 8-9 years in SWS.  

| **Maternal characteristics** |  |  |
| --- | --- | --- |
|  | n |  |
| Age at delivery (years) | 2143 | 30.6 (3.9) |
| Parity (≥ primiparous) | 2140 | 1067 (49.9) |
| Educational level (≥ A level) | 2136 | 1202 (56.3) |
| Smoked during pregnancy | 1984 | 358 (18.0) |
| Height | 2131 | 163.0 (6.5) |
| Pre-pregnancy weight | 2126 | 67.1 (14.0) |
| Pre-pregnancy BMI | 2124 |  |
| < 18.5 (Underweight) |  | 38 (1.8) |
| 18.5 - 25 (Normal) |  | 1221 (57.5) |
| 25 - 30 (Overweight) |  | 562 (26.5) |
| > 30 (Obese) |  | 303 (14.3) |
| Hours/week of strenuous physical activity (>0) | 2126 | 1379 (64.9) |
|  |  |  |
| **9 year offspring characteristics** |  |  |
|  | n |  |
| Sex (male) | 2140 | 1124 (52.5) |
| Height | 202 | 134.1 (6.0) |
| Weight | 202 | 30.8 (6.4) |
| Data are mean (SD), median (IQR) or number (%). |  |  |

**Supplementary Table 3b:** Baseline characteristics of mothers and children for those not undergoing offspring DXA at 9 years in ALSPAC. 

| **Maternal characteristics** |  |  |
| --- | --- | --- |
|  | N=7968 |  |
| Age at delivery (years) | 321 | 29.24 (4.39) |
| Parity (≥ primiparous) | 325 | 182 (56%) |
| Educational level (≥ A level) | 327 | 87 (27%) |
| Smoked during pregnancy | 296 | 42 (14%) |
| Height | 294 | 163.58 (6.68) |
| Pre-pregnancy weight | 294 | 61.38 (10.05) |
| Pre-pregnancy BMI | 302 | 22.96 (3.78) |
| Regular physical activity at least once a week | 313 | 215 (69%) |
|  |  |  |
| **9-year offspring characteristics** |  |  |
|  | N |  |
| Sex (male) | 99 | 48 (48%) |
| Height | 336 | 138.95 (6.08) |
| Weight | 336 | 34.31 (8.48) |
| Data are mean (SD), median (IQR) or number (%). |  |  |

**Supplementary Table 3:** Associations between offspring 3-year C-DII and offspring bone outcomes at 8-9 years in the SWS or ALSPAC.

|  | **3-year E-DII (units)** |  |  |  |  |  |  |  |  |
| --- | --- | --- | --- | --- | --- | --- | --- | --- | --- |
|  | Unadjusted |  |  |  |  | Adjusted ^1^ |  |  |  |
| **SWS** | n | β | SE | p |  | n | β | SE | p |
| BA (cm^2^) | 969 | **-11.72** | **4.79** | **0.02** |  | 950 | **-11.54** | **4.95** | **0.02** |
| BMC (g) | 969 | -5.75 | 3.66 | 0.12 |  | 950 | **-7.24** | **3.75** | **0.05** |
| aBMD (g/cm^2^) | 969 | 0.001 | 0.002 | 0.54 |  | 950 | -0.0003 | 0.002 | 0.86 |
| BMC for BA (g) | 969 | 1.76 | 1.99 | 0.38 |  | 950 | 0.16 | 1.96 | 0.94 |
|  |  |  |  |  |  |  |  |  |  |
|  | Unadjusted |  |  |  |  | Adjusted ^1^ |  |  |  |
| **ALSPAC** | n | β | SE | p |  | n | β | SE | p |
| BA (cm^2^) | 5710 | -2.75568 | 1.66481 | 0.098 |  | **4612** | **-4.74** | **1.79** | **<0.01** |
| BMC (g) | 5710 | -3.18894 | 1.86751 | 0.088 |  | **4612** | **-5.58** | **2.00** | **<0.01** |
| aBMD (g/cm^2^) | 5710 | -0.00081 | 0.00056 | 0.148 |  | **4612** | **-0.0015** | **0.0006** | **0.011** |
| BMC for BA (g) | 5710 | -0.17047 | 0.40611 | 0.675 |  | 4612 | -0.39 | 0.46 | 0.39 |

Table shows regression coefficient and standard error from univariable and multivariable linear regression analyses. Outcomes are whole body measurements, without heads. Results with p≤0.05 shown in bold. 

^1 ^Adjusted for offspring sex and age at DXA and maternal age at childbirth, educational level, pre-pregnancy BMI, parity, physical activity level and smoking in pregnancy status. 

**Supplementary Table 4:** Associations between maternal late pregnancy (34 weeks) and offspring bone outcomes at 9 years in the SWS or ALSPAC, with additional adjustment for offspring height.

|  |  | **Late pregnancy E-DII (units)** |  |  |  |
| --- | --- | --- | --- | --- | --- |
|  |  | Adjusted ^1^ |  |  |  |
| **SWS** |  | n | β | SE | p |
| BA (cm^2^) |  | 917 | -3.33 | 3.20 | 0.30 |
| BMC (g) |  | 917 | -2.66 | 1.91 | 0.17 |
| aBMD (g/cm^2^) |  | 917 | -0.0003 | 0.001 | 0.78 |
| BMC for BA (g) |  | 917 | -0.52 | 1.24 | 0.67 |
|  |  |  |  |  |  |
|  |  |  |  |  |  |
|  |  | Adjusted ^1^ |  |  |  |
| **ALSPAC** |  | n | β | SE | p |
| BA (cm^2^) |  | 4932 | -1.90 | 0.71 | **<0.01** |
| BMC (g) |  | 4932 | -2.65 | 0.90 | **<0.01** |
| aBMD (g/cm^2^) |  | 4932 | -0.0009 | 0.0004 | **<0.01** |
| BMC for BA (g) |  | 4932 | -0.57 | 0.33 | 0.0850 |

Table shows regression coefficient and standard error from multivariable linear regression analyses. Outcomes are whole body measurements, without heads. Results with p≤0.05 shown in bold. 

^1^ Adjusted for offspring sex, height and age at DXA and maternal age at childbirth, educational level, pre-pregnancy BMI, parity, physical activity level and smoking in pregnancy status. 

**Supplementary Table 5:** Associations between maternal late pregnancy (34 weeks) and offspring bone outcomes at 8-9 years in the SWS or ALSPAC, with additional adjustment for offspring weight.

|  |  | **Late pregnancy E-DII (units)** |  |  |  |
| --- | --- | --- | --- | --- | --- |
|  |  | Adjusted ^1^ |  |  |  |
| **SWS** |  | n | β | SE | p |
| BA (cm^2^) |  | 915 | -4.54 | 3.28 | 0.17 |
| BMC (g) |  | 915 | **-4.02** | **2.06** | **0.05** |
| aBMD (g/cm^2^) |  | 915 | -0.0008 | 0.001 | 0.47 |
| BMC for BA (g) |  | 915 | -1.11 | 1.29 | 0.39 |
|  |  |  |  |  |  |
|  |  | Adjusted ^1^ |  |  |  |
| **ALSPAC** |  | n | β | SE | p |
| BA (cm^2^) |  | 4932 | -0.62 | 0.72 | 0.39 |
| BMC (g) |  | 4932 | -1.16 | 0.84 | 0.17 |
| aBMD (g/cm^2^) |  | 4932 | -0.0006 | 0.0003 | 0.10 |
| BMC for BA (g) |  | 4932 | -0.48 | 0.33 | 0.15 |

Table shows regression coefficient and standard error from multivariable linear regression analyses. Outcomes are whole body measurements, without heads. Results with p≤0.05 shown in bold. 

^1^ Adjusted for offspring sex, weight and age at DXA and maternal age at childbirth, educational level, pre-pregnancy BMI, parity, physical activity level and smoking in pregnancy status. 

**Supplementary Figure 2:** Meta-analysis of associations between **childhood 3-year C-DII** and 9 year bone outcomes in the SWS and ALSPAC cohorts.**A) BA**

**B) BMC**

**C) aBMD**

**D) BMC adjusted for BA**
